# Novel hair snare and genetic methods for non‐invasive bobcat detection

**DOI:** 10.1002/ece3.8435

**Published:** 2022-01-24

**Authors:** Thomas F. Rounsville, Richard E. Rogers, Amy B. Welsh, Christopher W. Ryan, James T. Anderson

**Affiliations:** ^1^ School of Natural Resources West Virginia University Morgantown West Virginia USA; ^2^ West Virginia Division of Natural Resources Romney West Virginia USA; ^3^ West Virginia Division of Natural Resources Morgantown West Virginia USA; ^4^ Present address: James C. Kennedy Waterfowl and Wetlands Conservation Center Belle W. Baruch Institute of Coastal Ecology and Forest Science Clemson University P.O. Box 596 Georgetown South Carolina 29442 USA

**Keywords:** bobcat, hair snares, *Lynx rufus*, mtDNA, qPCR, West Virginia (USA)

## Abstract

Over the past 20 years, the use of non‐invasive hair snare surveys in wildlife research and management has become more prevalent. While these tools have been used to answer important research questions, these techniques often fail to gather information on elusive carnivores, such as bobcats (*Lynx rufus*). Due to the limited success of previous bobcat studies using hair snares which required active rubbing, this technique has largely fallen out of use, in favor of camera trapping. The goal of our study was to construct a novel, passive bobcat hair snare that could be deployed regardless of terrain or vegetation features, which would be effective for use in capture–recapture population estimation at a large spatial scale. This new hair snare was deployed in 1500 10‐km^2^ cells across West Virginia (USA) between two sampling seasons (2015–2016). Collected hair samples were analyzed with newly developed mitochondrial DNA primers specifically for felids and qPCR to determine species of origin, with enough sensitivity to identify samples as small as two bobcat hairs. Over the two years of the study, a total of 378 bobcat detections were recorded from 42,000 trap nights of sampling, for an overall rate of 0.9 detections/100 trap nights—nearly 2–6 times greater than any previous bobcat hair snare study. While the overall number of recaptured animals was low (*n* = 9), continued development of this platform should increase its usefulness in capture–recapture studies.

## Graphical abstract

Gathering large‐scale population information on elusive carnivores, like bobcats, can be difficult. We developed a novel hair snare to non‐invasively sample bobcats for use in capture–recapture studies at a large spatial scale. In addition, we designed felid‐ and bobcat‐specific qPCR primers to assist in determining the species of origin of collected hair samples.

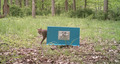

## 1 INTRODUCTION

The bobcat (*Lynx rufus*) is a moderately sized felid with established populations across the contiguous United States, except for the state of Delaware, making it one of the most widely distributed carnivores in North America (Roberts & Crimmins, 2010). In some states, bobcats are currently hunted and trapped for their valuable fur; however, in others, they are considered a protected species due to either unknown or low overall abundance, or lack of public acceptance of any type of harvest. According to a survey of state wildlife agencies conducted by Roberts and Crimmins (2010), bobcat abundance across the United States has increased dramatically since the 1980s. However, only about 50% of the responding state wildlife agencies reported an estimated population size or density estimate for their bobcat populations. Bobcats are not provided any protections under United States federal law, but their harvests are governed by the Convention on International Trade in Endangered Species of Wild Fauna and Flora (CITES) under Appendix II, due to their similarity to the endangered Iberian lynx (*Lynx pardinus*; United States Fish & Wildlife Service, 1982). To maintain compliance with CITES, any state that allows hunting or trapping of bobcats, like West Virginia (USA), is required to demonstrate the sustainability of these activities, which may or may not require detailed demographic information. Since bobcats are an elusive carnivore species, the collection of these data can be a difficult task.

Camera traps, scat transects, and hair snares are non‐invasive methods commonly used to collect bobcat demographic information. Camera traps have been successfully used to document bobcat presence and estimate abundance in several studies, but only when the unique spot patterns of individuals can be identified (Clare et al., 2015; Comer et al., 2011; Heilbrun et al., 2006; Larrucea et al., 2007; Symmank et al., 2008; Thornton & Pekins, 2015). Camera trap costs have declined significantly since the first study that used cameras to identify bobcat individuals based on fur spot patterns in 2003 (Heilbrun et al., 2003) in Texas, USA; however, using cameras to survey large areas still requires a sizable investment of resources. Additionally, it is not possible to accurately identify individual animals by spot patterns across much of the bobcat's range, particularly in West Virginia, and other surrounding northeastern states (Croteau et al., 2012; Morin et al., 2018; Young, 1958). Scat transects, conducted using both human observers (Morin et al., 2018; Ruell et al., 2009) and detection dogs (Harrison, 2006; Long et al., 2007), have also been successfully applied to the estimation of bobcat abundance or occupancy. When directly comparing these two methods, scat detection dogs were the most effective at finding bobcat samples, but they also had the greatest associated costs per sample collected (Harrison, 2006; Long et al., 2007), which can greatly reduce the area that can be surveyed with limited resources. In contrast, hair snares have the lowest deployment costs, allowing for a much larger area to be surveyed for the same resource expenditure (Harrison, 2006); however, this may not always be the case once the costs of genetic analyses are considered.

The use of hair snares in wild felid research came into prominence after the development of the carpet scratch pad by McDaniel et al. (2000), which was successful in collecting hair samples from Canada lynx (*Lynx canadensis*). This device was effectively used to study other species such as ocelot (*Leopardus pardalis*; Weaver et al., 2005); however, little success was reported when using the same carpet scratch pads to specifically target bobcats. These same carpet scratch pads were deployed for bobcat sampling in multiple US states: New Mexico (Harrison, 2006), Vermont (Long et al., 2007), and Texas (Comer et al., 2011), with one, zero, and one bobcat detection recorded, respectively. The authors of these three studies reached the same conclusion: Hair snares are not successful at non‐invasively sampling wild bobcats. More recently, the efficacy of the original carpet scratch pads for Canada lynx research has also come into question due to the variability between individuals in cheek rubbing behaviors required for sampling (Crowley & Hodder, 2017).

Due to the poor performance of carpet scratch pads for bobcat research, a more recent study has utilized a new approach to attempt to collect bobcat hair. In the upper peninsula of Michigan, USA, Stricker et al. (2012) deployed a modified cable snare (DePue & Ben‐David, 2007) that was designed as a single‐sample device placed in distinct travel corridors surrounding sites baited with deer carcasses. This device was constructed specifically as a passive device to collect hair samples without the required cheek rubbing behavior of the carpet scratch pad. Over the course of an 8‐week study in a 278.5 km^2^ study area, a total of 230 hair samples were collected, of which 17 originated from bobcats. This study documented the successful use of hair snares to sample wild bobcats and estimate population size by using a novel method. Kautz et al. (2019) also successfully used this same snaring method to estimate bobcat density in a capture–recapture study that also took place in the upper peninsula of Michigan.

An additional difficulty that researchers face when utilizing non‐invasive methods is the scarcity of modern genetic analysis methods created specifically for samples with low DNA quantity and quality. Previous non‐invasive felid studies identified species of origin by amplifying 300–1000 nt fragments of mitochondrial DNA (mtDNA) using universal primers followed by restriction enzyme digest or DNA sequencing (Foran et al., 1997; Mills et al., 2000; Weaver et al., 2005). Non‐invasively collected samples often yield low quantity and low‐quality DNA, and more recently, Stricker et al. (2012) and Kautz et al. (2019) utilized smaller DNA fragments since these degrade more slowly when exposed to the environment. However, even with the use of smaller DNA amplicons, a high percentage of collected hair samples still fail in DNA sequencing when using traditional methods.

Although qPCR is not a new innovation, its implementation in wildlife studies has been slow and uneven. However, qPCR has advantages over traditional PCR followed by capillary sequencing for species identification of hair samples. Firstly, qPCR can be used to detect a species of interest in a mixed‐species sample by designing species‐specific primers. This contrasts with the discarding of mixed‐species samples in studies using the traditional methodology of visual inspection or light microscopy for species identification, even though it may result in the disposal of valid samples from the species of interest. Second, qPCR assays can be designed to amplify smaller fragment sizes than those needed for capillary sequencing, while still providing enough information for species identification.

Our research project was completed as a portion of a study seeking to estimate the relative abundance and, potentially, the density of bobcats across West Virginia (63,000 km^2^), to aid in management decisions on the species. Hair snares were selected as the only non‐invasive method that would be economically feasible to sample such a large area. While the technique used by Stricker et al. (2012) and Kautz et al. (2019) was successful in collecting hair samples from bobcats in the Upper Peninsula of Michigan, it would likely not work well in West Virginia. The topographic complexity of West Virginia, the lack of distinct travel corridors between patches of forest separated by agricultural areas, and the massive amount of bait that would be required rendered the Stricker et al. (2012) method logistically prohibitive for our research project. Thus, the first goal of our study was to develop a new and effective hair snare that was able to meet the following criteria: (1) can be deployed independent of terrain features (i.e., nearby trees, rock formations, travel corridors, or other vegetation); (2) does not require behavioral cheek rubbing to collect samples; (3) is easy to transport, deploy, and check; (4) is durable enough for long‐term use (6+ months deployed continuously); and (5) reliably collects hair samples from individual bobcats. Our second goal was to develop a new felid‐specific quantitative PCR (qPCR) method for evaluating the species of origin of collected hair samples. Our third goal was to evaluate the success of the new hair snare in terms of detections per 100 trap nights and compare to previous studies. Our fourth and final goal was to evaluate the bycatch of our newly developed hair snare.

## 2 STUDY AREA

The study area for our research project was the entire U.S. state of West Virginia, which is topographically complex throughout and ranges in elevation from 100 to 1300 m. Forests were the most prominent cover type and comprised 78% of the total study area, with oaks (*Quercus* spp.), maples (*Acer* spp.), hickories (*Carya* spp.), and yellow poplar (*Liriodendron tulipifera*) being the most prominent tree species (Randall et al., 2016). Higher elevation areas in the eastern portions of the state were instead dominated by conifers such as eastern hemlock (*Tsuga canadensis*), red spruce (*Picea rubens*), and eastern white pine (*Pinus strobus*). Averaged across the state for the years of this study (2015 and 2016), the mean maximum temperature in July was 28.4°C, in contrast to 3.1°C in January, and the mean annual precipitation was 119.4 cm (National Oceanic & Atmospheric Administration, 2021).

## 3 METHODS

### 3.1 Hair snare cubby design

**FIGURE 1:**
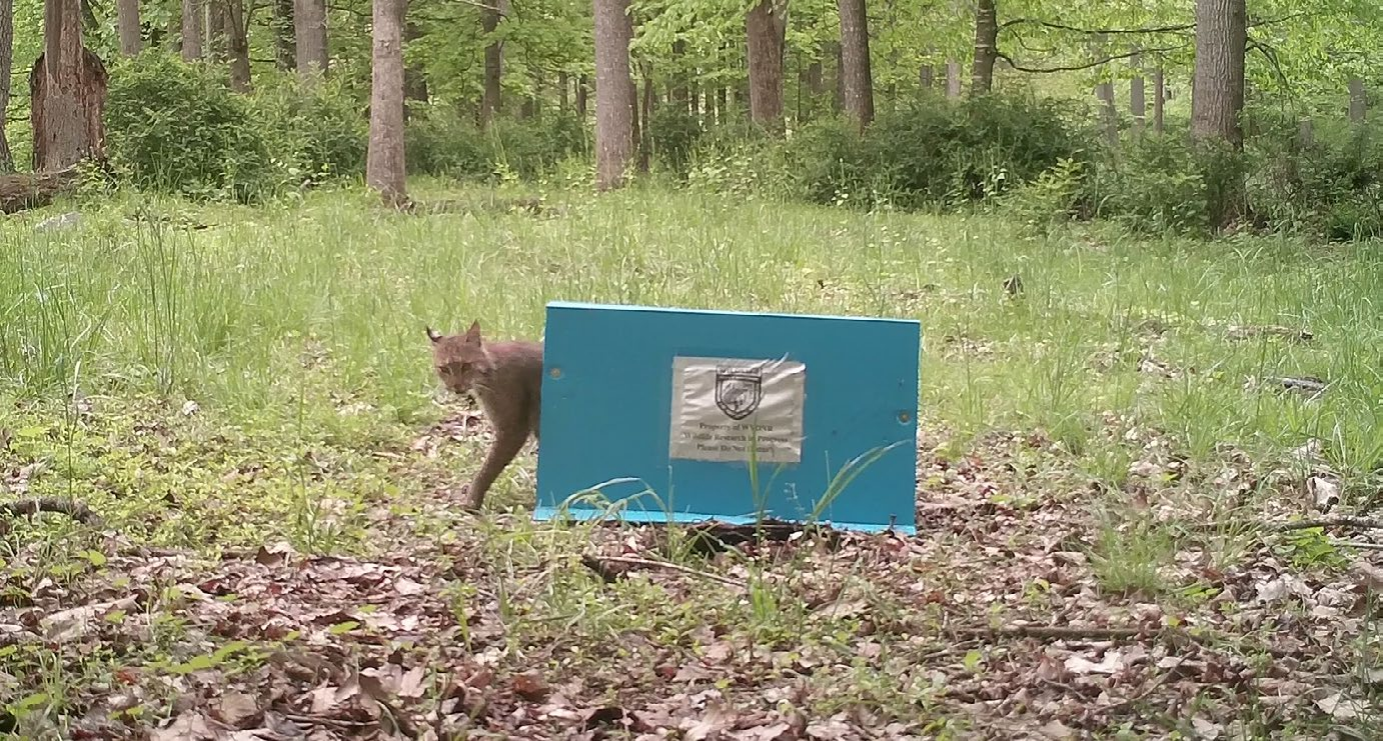
Bobcat photographed by a remote camera as it walks through a hair snare cubby and a hair sample is collected

**FIGURE 2:**
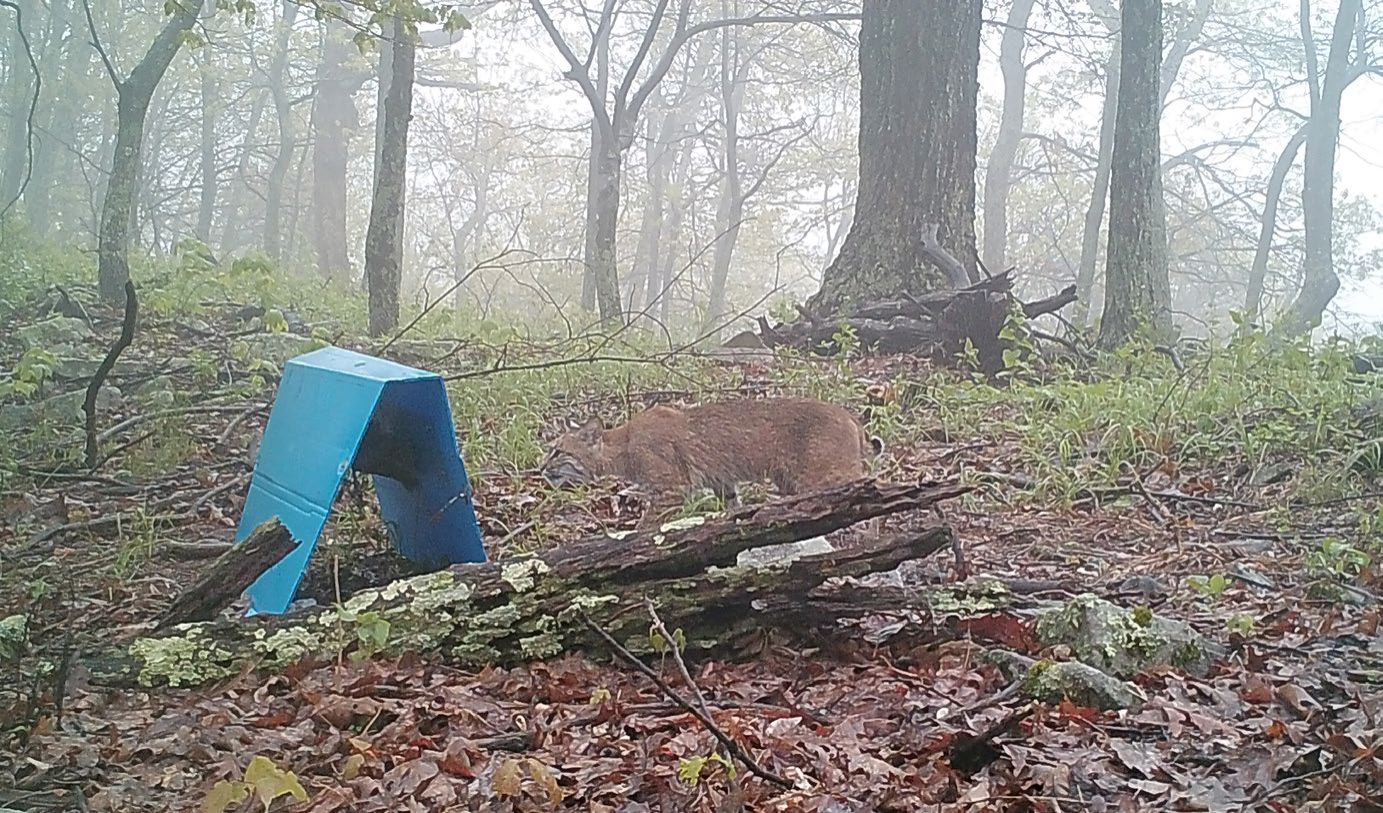
Bobcat photographed by a remote camera as it cautiously investigates a hair snare cubby in a headfirst posture

To meet the specific needs of sampling bobcats in the central Appalachian Mountains, we developed a new hair snare using what was described by Kendall et al. (2008) for sampling fisher (*Pekania pennanti*) populations as a starting point for modifications. We increased the overall height and length of the device to accommodate bobcat stature. Secondly, we did not add wire mesh to block one of the entranceways so bobcats could walk through the entire sampling device (Figure 1). Thirdly, we staggered the sampling gun brushes located in the entranceways to allow for the collection of samples from bobcats that either entered the device entirely, or only placed their heads in the device (Figure 2). For the remainder of the manuscript, this device will be referred to as the hair snare cubby, or simply abbreviated as cubby. Specific considerations that contributed to the final design of the device were the appeal of the cubby to bobcats, the ability to collect a hair sample without behavioral rubbing, the ease of deploying the cubby, and the ability to set up the cubby without requiring specific vegetation or terrain features.

**FIGURE 3:**
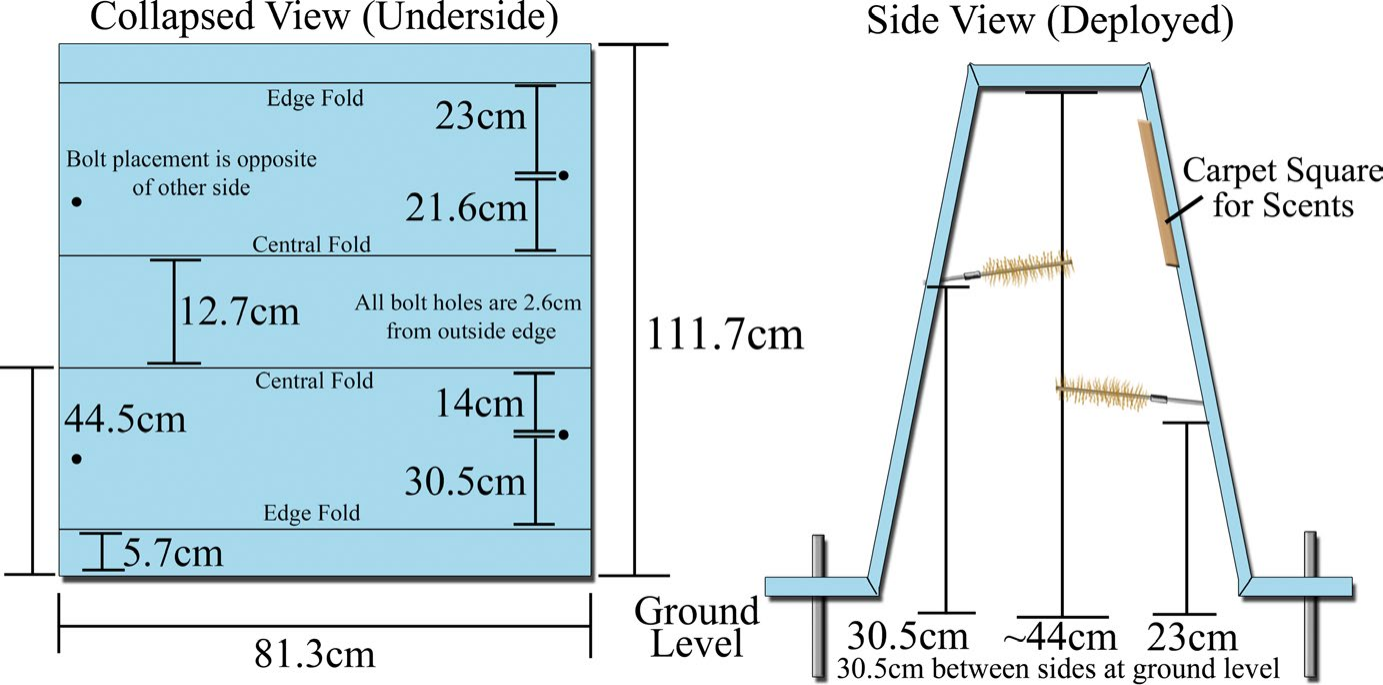
Bobcat hair snare cubby construction schematic. The black dots on the collapsed view diagram indicate where perforations were made to affix the #8–32 bolts to which the gun brushes were attached. The bolt placement was staggered such that each lateral side of the cubby had one entrance where the bolt placement was low and one that was high. All bolt perforations were made at a distance of 2.6 cm from the outside edge of the sampling device. Solid black lines indicate where creases were made by folding the corrugated plastic against a solid object with a right angle, such as a laboratory benchtop. To create the lip needed for affixing the ground spikes, a second set of creased folds at the edge were required, on the opposite side of the corrugated plastic sheet as those used for the central folds. When erecting the cubby, spikes were driven through the four outside corners of the device at locations roughly 2.6 cm from any outside edges

We constructed each hair snare cubby from a 111.7 cm × 81.3 cm pad of blue corrugated plastic of 4.8 mm thickness (Figure 3). Rather than choose a color that blended into the surrounding terrain, we purposely chose blue for the corrugated plastic since it is a possible visual attractant for felids (Loop et al., 1979). We bent the plastic pads into a trapezoidal shape on the corner of a laboratory bench such that the flat apex of the cubby would stand roughly 44 cm from the ground—high enough for the average bobcat to enter without crouching. To each cubby, we affixed four 5.1 cm long #8–32 bolts which we placed 2.6 cm from the outside edge and staggered either 14.0 or 21.6 cm from the nearest apex crease. We then secured these bolts tightly using two sets of oversized fender washers and nylon‐centered locking nuts. Once attached, we bent the bolts inward toward the center of the cubby and parallel to the ground so that the tip of the bolt was roughly 2.6 cm from the side wall of the cubby. Next, we threaded coupling nuts of #8–32 thread and length 1.6 cm onto the bolts to serve as the attachment point for the gun brushes used in sampling. Finally, we attached a 20.3 cm × 20.3 cm square piece of brown outdoor carpet to the upper middle of the inside surface of each cubby using roughly 10 staples of approximately 9.5 mm in length. Supplies required for the construction and equipping of one cubby were roughly $25 USD.

### 3.2 Sampling design

To gather representative samples over such a large study area, a map of West Virginia was overlaid with a grid of 10‐km^2^ cells. We chose this cell size based on the recommendations of White (1982) that at least two devices should be placed within the smallest possible home range for greater accuracy in capture‐recapture studies. Female bobcats generally have smaller home ranges than males (Lovallo & Anderson, 1996), and their average home range size in West Virginia at the beginning of this study was thought to be ~20 km^2^, based on work completed in similar habitats of neighboring Virginia, USA (McCord & Cardoza, 1982; Progulske, 1952). We selected clumped, contiguous groups of 25 equally sized cells (5 × 5 square) for study based on several criteria: (1) <25% of the cells in a group contained an urban area; (2) cell clusters with a diversity of habitat types were preferred over those that were dominated by a single habitat type (e.g., forest); (3) equal sampling effort between the 6 distinct ecological regions of West Virginia as defined by Uhlig and Wilson (1952); (4) remote, inaccessible areas were avoided due to logistical constraints; and (5) clumped sites were spaced as evenly as possible throughout the state to reduce the error of future interpolations of bobcat occupancy and abundance.

### 3.3 Field sample collection

**FIGURE 4:**
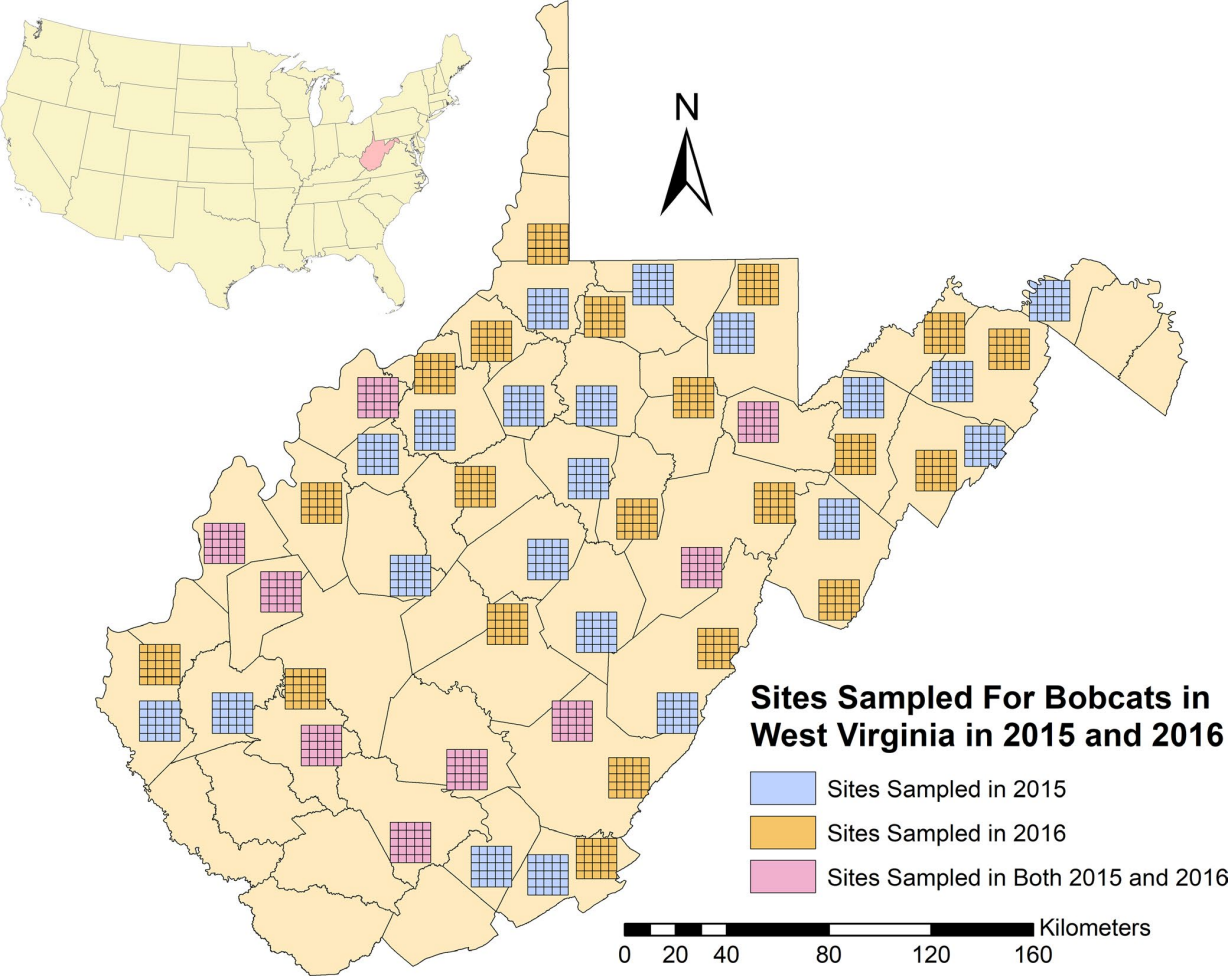
Map of the sampled study sites in West Virginia (USA) during the 2015 and 2016 field sampling seasons. A single hair snare cubby was placed in each of the cells of the 5 × 5 study site during sampling. Sampling took place at each study site over the course of a 4‐week session. For both 2015 and 2016, a total of 5 sampling sessions were undertaken between March and July. A total of six 5 × 5 study sites were sampled simultaneously during the same session, with a total of 30 sites (7500 km^2^) being sampled in 2015 another 30 in 2016

During the 2015 and 2016 field seasons, we sampled 30 individual 250 km^2^ study sites (25 contiguous 10‐km^2^ cells in a 5 × 5 configuration), for a total sampled area of 15,000 km^2^ across both years (Figure 4), under West Virginia University IACUC Protocol #14‐1108. We sampled 9 of the same site locations in both 2015 and 2016 to assess the usefulness of this method in potential multi‐year studies. Within each of the 25 cells at a study site, we placed one hair snare cubby over a 4‐week session, for a total of 700 trap nights. In each of the 6 ecological regions, one study site (5 × 5 grid) was sampled during each session. A total of 5 sampling sessions took place during March–August of 2015 and March–July of 2016 (Table 1). Cubby placement inside of each cell was opportunistic based on landowner approvals as well as available habitat and terrain types. When available, we selectively placed cubbies on or adjacent to game trails or dirt roads located within forested habitats with nearby edge, as recommended by Clare et al. (2015), for maximizing bobcat detection. However, we deployed many cubbies in suboptimal locations or habitats due to extraneous circumstances, or to provide useful habitat selection data for subsequent occupancy modeling (Rounsville, 2018).

**TABLE 1:** Sampling dates for each session for both the 2015 and the 2016 sampling seasons

Session number	2015	2016
Session 1	3/23/15–4/20/15	3/2/16–3/30/16
Session 2	4/21/15–5/19/15	3/31/16–4/28/16
Session 3	5/20/15–6/17/15	4/29/16–5/20/16
Session 4	6/18/15–7/16/15	5/31/16–6/28/16
Session 5	7/17/15–8/14/15	6/29/16–7/27/16

Once a site was selected for cubby deployment, we used a trowel to dig a 7.62‐cm deep hole under what would become the center of the cubby. In this hole, we placed 4 g of Caven's Minnesota Brand Bobcat Chunk Bait (Minnesota Trapline Products, Pennock, MN, USA) as a food enticement for animals to enter the sampling device. Next, we added a handful of pillow stuffing to the same hole and arranged it as a visual attractant to look like the nest of a rodent. We then oriented the cubby on top of the mimic cache hole and secured one long side to the ground using two 30.5‐cm landscaping spikes driven directly through the edge flaps, roughly 2.5 cm from any outside edge. After securing one side of the cubby, we flipped the long edge over top of the anchored spikes and exposed the carpet that was attached to the upper inside surface of the cubby.

Along the top half of the carpet square when the cubby was erected, we smeared 0.5 g of Light Skunk paste calling lure (Cage Magic Lures, Barstow, California, USA). On the bottom half, we added 7 ml of a scent lure in an “S” pattern. This lure was based on the recommendations of McDaniel et al. (2000) and was a mixture of 3800 ml of Beaver Castoreum Tincture (Kaatz Bros. Lures, Savanna, IL, USA), 250 ml glycerin, 250 ml propylene glycol, and 250 ml of Imitation Catnip Oil (Kaatz Bros. Lures). To each of the #8–32 bolts inside of the cubby, we attached a 0.30 caliber gun brush using a 1.6 cm length threaded joining nut. We then erected the cubby such that the distance between the bottom of the 2 inside edges of each opening was 30.5 cm, using a landscaping spike as a guide. We drove 2 additional 30.5‐cm landscaping spikes through the unsecured side to firmly attach the cubby to the ground. As an additional visual attractant, we hung a compact disk via roughly 61 cm of colored flagging tape from a nearby tree with long sight distance. Finally, we deployed camera traps (Moultrie #MCG‐12693 in 2015 and 2016 and Browning #BTC‐7FHD in 2016 only) in video mode to record bobcat interactions with cubbies at a small number of sites where we considered bobcat encounters to be likely.

We checked deployed cubbies every seven days and collected all four gun brushes in the same labeled manila envelope, pooling the hairs together from all four gun brushes and processing as a single sample. During the weekly checks, we replaced scent baits and lures along with the gun brushes. Following the second week of data collection, we moved the cubbies roughly 15 m to alleviate a potential trap avoidance response (Kendall et al., 2008). We removed any unconsumed baits and visual attractants from the old site and filled the previous cache hole with earth. At the conclusion of each four‐week sampling session, we removed all cubbies from sampling locations and reused them in a new study site.

### 3.4 Hair analysis

We examined gun brushes collected in the field under a dissecting microscope for the presence of animal hair. We morphologically assigned the putative species of hair samples using the hair identification keys and resources of Hairdatabase.com (Knecht et al., 2015). We then removed all snared hairs from the four gun brushes considered to be a single sample using a 70% ethanol cleaned forceps and placed them into a single 1.5‐ml microcentrifuge tube containing 100–125 µl of molecular grade distilled water. We retained both hair shafts and hair follicles to ensure that as much DNA as possible was available for genetic analyses and stored hair samples at −20°C until DNA extraction procedures.

We extracted the DNA from the collected hair samples with a Qiagen DNeasy 96 Blood & Tissue Kit and a modified protocol to accommodate the hair samples. To each tube containing hair samples and water, we added 20 µl of proteinase K, 30 µl of 1 M DTT (dithiothreitol), and 250 µl of buffer ATL. We then incubated the samples at 56°C on a slowly rotating platform between 5 and 12 h until the hairs completely dissolved. For the remainder of the DNA extraction procedures, we followed the manufacturer's suggested protocol except we used two 100 μl final elution steps. Following elution, we dehydrated the 96‐well extraction plates in a vacuum centrifuge at 60°C until roughly 75 μl of buffer AE remained in each well of the plate (roughly 6 h) to further concentrate the DNA.

To reduce the loss of collected data as much as possible, we developed a sensitive new two‐step qPCR species identification method specifically for non‐invasively collected bobcat and domestic cat samples of low quality. We designed primers by creating alignments of mtDNA sequences obtained from the United States National Center for Biotechnology Information (NCBI) GenBank in the program BioEdit 7.0.5.3 (Hall, 1999). We aligned the mtDNA sequences for the following species with the respective NCBI GenBank accession numbers: bobcat (GQ979707.3), domestic cat (U20753.1), black bear (*Ursus americanus*; KM257060.1, AF303109.1), domestic dog (*Canis familiaris*; U96639.2, AY729880.1), red fox (*Vulpes vulpes*; JN711443.1, KP342452.1), gray fox (*Urocyon cinereoargenteus*; KP129097.1, KP129108.1), white‐tailed deer (*Odocoileus virginianus*; HQ332445.1), Virginia opossum (*Didelphis virginiana*; Z29573.1), raccoon (*Procyon lotor*; AB297804.1), fisher (*Pekania pennanti*; HQ705180.1, HM106327), and human (*Homo sapiens*; GU170821.1; GU170815.1) and scanned specifically for regions where the domestic cat and bobcat sequences diverged significantly from the other species. We designed a first primer pair (FelidV‐F: 5′‐CCTATTTAACCTACCACACCCACAAG‐3′ and FelidV‐R:5′‐GCCAGATGCTTTGTTTAAGCTACATC‐3′) specifically to amplify a 105 nt fragment of mtDNA from most wild felids native to, or commonly found in, North America. We designed a second primer pair (LynX‐F: 5′‐GCAAACATCAGCACCTCCGTT‐3′ and LynX‐R: 5′‐CTAGTAGGGTGAAGACGTAGGCTTG‐3′) to amplify a 109 nt fragment of mtDNA from only two felid species native to North America—the bobcat and Canada lynx.For the first part of our two‐step species identification procedure, we used primer set FelidV to determine which hair samples were derived from a felid source, followed by LynX to identify which samples originated from bobcats. Since the study area is not located within the known range of the Canada lynx, any sample which produced a PCR product for primer set LynX was concluded to have been collected from a bobcat. We used a two‐part species determination process because SYBR green assays can have a false‐positive rate higher than other genetic methods, due to their increased sensitivity, and the fact that SYBR green intercalates with any dsDNA, not just a desired amplification product. We also validated the specificity of both primer sets by attempting to amplify DNA extracts of the mammals native to West Virginia most likely to be sampled by a cubby including: black bear, domestic cat, fisher, opossum, raccoon, woodchuck (*Marmota monax*), and white‐tailed deer.

We used identical PCR conditions and reagents for both primer pairs: 5 μl of (2×) Bio‐Rad iQ SYBR Green SuperMix, 1 μl of nuclease‐free distilled water, 0.5 μl each of the appropriate 2 primers (10 μM concentration stock, 0.5 μM in solution), and 3 μl of DNA template, for a 10 μl total reaction size. We completed our PCRs on a Bio‐Rad CFX Connect Touch Real‐Time PCR Detection System with the following conditions: 95°C for 3 min followed by 40 cycles of 95°C for 10 s and 60°C for 30 s with a quantification image taken at the conclusion of each step. We considered samples to be positive for primer set FelidV and Lynx if the CQ (CT) values were ≤33.5 and ≤32.5, respectively. We determined these cutoff values by repeatedly analyzing positive control samples of two small bobcat hairs (~5 mm) without follicles cut from a tanned hide, which we determined to be the limit of detection of this assay that would amplify consistently. On each plate of hair samples analyzed, we included at least three replicates of our limit of detection bobcat hair positive controls along with no‐template controls (NTC) to ensure the consistent accuracy of these cutoff thresholds and discarded any plate where the expected CT values of these controls varied considerably from expected. We also used no‐template DNA extractions as negative controls to ensure sample contamination did not occur during DNA extraction procedures.

To evaluate the success of the hair snare cubby in collecting hair samples from the same bobcat across sampling weeks, we screened each sample that amplified both the FelidV and LynX mtDNA fragments with a three‐locus multiplex of highly heterozygous and polymorphic microsatellites with fragment sizes ≤150 bp (FCA008, FCA90, and FCA77; Menotti‐Raymond et al., 1997, 1999; Menotti‐Raymond & O’Brien, 1995). Previous population genetic research we completed on the West Virginia bobcat population revealed that the non‐exclusion probability of identity of these 3 loci was 2.279 × 10^−4^, with a p‐sib of 0.047 (Rounsville, 2018; Tables S1 and S2). This means that there is greater than a 95% chance that any two random bobcats from this population (including siblings) would have a different DNA profile at just these three loci. However, if any combination of only 2 of the 3 loci was successfully amplified, the probability of siblings having different DNA profiles was still greater than 86% (Table S2).

We completed each multiplex PCR in a 10 μl reaction volume which consisted of 5 µl of the 2× Qiagen Multiplexing Master Mix, 300 nmol of each individual primer, and 3.5 µl of DNA template. We used the following PCR conditions: 15‐min hot‐start at 95°C, 45 cycles of 94°C for 30 s, 57°C for 90 s, and 72°C for 90 s, with a final extension of 72°C for 10 min. We resolved amplified DNA fragments on a Beckman‐Coulter GeXP Genetic Analysis System (AB SCIEX) and recorded fragment sizes using the associated GeXP fragment analysis software.

We attempted to amplify each sample with two separate PCRs and removed samples failing to produce genotypes for at least two of the three loci from further analysis. We also removed samples that had variable genotypes across two or more PCRs, but two or less alleles at one locus, which was likely caused by allelic dropout. Finally, we removed samples that had more than 3 alleles at a single locus since we considered them to be mixed samples of multiple individuals. Next, we used GENECAP v1‐2 (Wilberg & Dreher, 2004) to find samples with matching genotypes. We amplified matching samples using additional microsatellites in two multiplexes, A (FCA23, FCA25) and C (FCA43, 6HDZ700, and FCA45; Menotti‐Raymond et al., 1997, 1999; Menotti‐Raymond & O’Brien, 1995; Williamson et al., 2002). Once we amplified a combination of loci for each sample pair where p‐sib <10^−3^, we considered samples still matching at all loci to be recaptures. We considered sample pairs that had a mismatch of ≥1 allele, to have originated from separate individuals.

## 4 RESULTS

Of the total 2014 hair samples collected in the 2015 sampling season, 1641 yielded a sample suitable for genetic analysis (i.e., containing ≥5 or more hairs, or a single hair with a follicle, any number of hairs with visible follicles, or samples of ≤5 hairs that even had vague microscopic similarity to felid hairs). In 2016, a total of 2108 hair samples were collected, of which 1301 were suitable for genetic analysis. Of the 6000 individual site‐week samplings that took place, 49% (2942/6000) yielded a hair sample suitable for qPCR. Our analysis using primer set FelidV revealed that in 2015, 26.7% (438/1641) of the collected hair samples were derived from a felid source, as compared to 25.9% (337/1301) in 2016. Subsequent analysis of these felid‐positive samples with primer set LynX determined that 278 (63.5%) and 100 (29.7%) of these samples originated from bobcats, for the 2015 and 2016 sampling seasons, respectively. The remaining felid‐positive samples that did not have a CQ ≤32.5 for primer set LynX were most likely collected from domestic cats. Since each study site was sampled for a total of 700 trap nights, and a total of 60 study sites were examined, the performance of the bobcat hair snare cubby was evaluated for 21,000 trap nights each for both the 2015 and 2016 seasons. The 2015 season produced 278 confirmed bobcat detections, for a rate of 1.32/100 trap nights. The 2016 sampling season produced 100 confirmed bobcat detections, for a rate of 0.47/100 trap nights. When considering both sampling seasons together, a total of 378 bobcat detections were recorded for 42,000 trap nights, a rate of 0.9 detections/100 trap nights (Table 2).

**TABLE 2:** Comparison of the detection rate performance of the bobcat hair snare cubby deployed in West Virginia in 2015 and 2016 sampling seasons with other hair snares specifically designed to sample bobcats

Hair snare type	Total bobcat detections	Trap nights	Bobcat detections/100 trap nights	Bobcat density estimate/100 km^2^	Location in USA
2015 season	278	21,000	1.320	>3.567 ± 0.235^a^	West Virginia
2015–2016 combined	378	42,000	0.900	>3.836 ± 0.191^a^	West Virginia
Cable snares^b^	269	47,616	0.565	3.73^b^	Michigan
2016 season	100	21,000	0.470	>4.104 ± 0.147^a^	West Virginia
Cable snares^c^	17	9016	0.189	3.0^c^	Michigan
Scratch pads^d^	0	2072	0.000	8.43–14.04^e^	Vermont
Scratch pads^f^	1	700	0.143	11.51–17.26^e^	New Mexico
Scratch pads^g^	1	1680	0.060	29.0^h^	Texas

Note

Bobcat density estimates from each sampled locality are also included.

^a^ Rounsville (2018).

^b^ Kautz et al. (2019).

^c^ Stricker et al. (2012).

^d^ Long et al. (2007).

^e^ Estimated statewide density calculated from bobcat population data provided by US states in Roberts and Crimmins (2010).

^f^ Harrison (2006).

^g^ Comer et al. (2011).

^h^ Symmank et al. (2008) calculated this density when simultaneously surveying the same study area with camera traps.

From the 378 samples that originated from bobcats, a total of 230 (60.8%) were successfully genotyped for at least two of the three loci of the initial sample screening multiplex (FCA008, FCA90, and FCA77), with 46.1% (106/230) producing genotypes at all 3 screening loci. Only a small number of samples (~3%) appeared to have been collected from mixed felid species, or multiple bobcat individuals, and were removed from the study. Duplicate genotypes were found for 18 samples with a p‐sib <0.001, even after the secondary microsatellite analysis, indicating that these samples were recaptures. Of the 230 sample genotypes, 212 bobcats were found to be captured once and 9 bobcats were captured twice. No bobcat was recaptured at the same site where it was originally captured. Also, 55.6% of all recaptures occurred during the same sampling week that an individual had already been captured at a nearby site, within three or less sampling cells (roughly 10 km) from the original capture location.

While a total of 26.3% (775/2942) of all samples were collected from felid sources, additional DNA tests to determine species of origin were not conducted on the remaining non‐felid samples to reduce overall project costs. However, putative species of origin was assigned to these samples during hair removal procedures from the gun brushes using microscopy techniques and the most common bycatch species were raccoon and opossum, which accounted for 44.6% (1312/2942) of collected samples suitable for genetic analysis. Another common bycatch species was black bear, which accounted for 7.9% (232/2942) of the total collected samples. Fisher, red fox, and gray fox were all detected as bycatch species; however, each comprised <1% of the total collected samples.

Combining both the 2015 and 2016 sampling seasons, we recorded a total of 195 incidents of cubbies being lost, damaged, destroyed, or tampered with over the course of a week of sampling. Roughly 75% (147/195) of these incidents were reports of cubbies being damaged but still available for bobcat sampling—although likely at a reduced capacity. Only 3.6% (7/195) of incidents were directly attributable to human causes. By far, black bears caused the most cubby incidents and accounted for 77.9% (152/195) of all instances of cubbies being lost, damaged, destroyed, or tampered with.

## 5 DISCUSSION

The bobcat hair snare cubby was successful in collecting hair samples from wild bobcats throughout West Virginia. When compared to other recent bobcat hair snare studies (Table 2), the bobcat hair snare cubby had roughly 2–6 times the detection rate per unit effort. However, additional testing is required to assess whether this improved detection rate was caused directly by the hair snare cubby design or other extraneous factors when comparing different study locations, such as differences in bobcat density or activity levels. Beyond the possible increased detection rates of the hair snare cubby as compared to other hair snaring methods, there are three additional benefits of using this device: (1) hair samples are collected on gun brushes inside of the cubby, and samples are not exposed to DNA‐damaging moisture or UV light (Kendall et al., 2008) until sample retrieval; (2) the bobcat hair snare cubby is not a single‐capture device, which is advantageous when collecting non‐invasive samples from elusive carnivores, since non‐target captures are common; and (3) the bobcat hair snare cubby is not dependent on nearby terrain features for setup and can be deployed with the same level of ease in dense forest as prairie.

While samples consisting of DNA from multiple individuals of the same species can cause genotyping errors (Pauli et al., 2008), single‐capture devices like those used by Stricker et al. (2012) run the risk of being triggered by more common species and then being unavailable to rarer ones, like bobcats. Since 44.6% of the hair samples we collected in this project appeared microscopically to have originated from raccoon or opossum, many bobcat captures would have been missed if our device was single‐use and was triggered and made unavailable by undesired species. We found that same site, same‐week sample mixtures of multiple bobcat individuals were rare, accounting for only 3% of the total number of samples that were successfully genotyped. However, it is possible that some samples comprised of multiple individuals were inadvertently misclassified as originating from a single animal due to the combination of allelic dropout and the use of a minimum of only 2 microsatellite loci for the preliminary screening of samples. Although this is unlikely, due to the high level of heterozygosity of the loci used (>70%) and our sampling strategy which took into account bobcat home range size. While additional work is required to determine whether this occurs in other localities, but at least in West Virginia, multiple bobcats being sampled by the same cubby during the same week is a rare event when using one device per 10‐km^2^ cell. Thus, we are of the opinion that single‐sample devices are not required when using hair snares to sample bobcats in similar ecotypes and sampling design as to those in our study.

Although the design and deployment of the bobcat hair snare cubby successfully met 4 of the 5 subpoints of this project's first objective, this device did not reliably collect hair samples from individual bobcats in all situations. At the absolute minimum, we sampled 212 individual bobcats with the hair snare cubby, which suggests that the exploratory behaviors resulting in animals being sampled are common—or at least much more common than the rubbing behaviors on which the earlier underperforming hair snare designs were based (Comer et al., 2011; Harrison, 2006; Long et al., 2007). However, we only captured 9 bobcat individuals more than once, and none at the same site, which shows that this method, in its current state, is not ready for wide‐scale deployment in capture–recapture population studies. A low bobcat recapture rate is not unique to our study, since Stricker et al. (2012) also experienced a low recapture rate.

While we have not yet been able to directly evaluate the causes of this lack of recaptures in a follow‐up study, our hypothesis is that most bobcats are only attracted to the cubbies when they provide a novel stimulus. Throughout our study, we used the same scent baits and lures, to which the bobcats may have been accustomed, and became disinterested during subsequent exposures. Evidence to support this hypothesis was collected in a new bobcat occupancy study in New Jersey, USA, which used the hair snare cubby paired with cameras (Personal Communication: Anthony McBride, New Jersey Division of Fish and Wildlife). During this study, scent types were changed weekly, based on our suggestion, and bobcats remained interested in the cubbies throughout the entire sampling period. In addition, this same study found that sites where cameras were paired with cubbies recorded more bobcat photographs and videos than sites with just a camera and scents, possibly because bobcats used cubbies as a visual stimulus to focus their search for the source of a scent lure.

Additional evidence from our own study that supports our hypothesis that bobcats are only attracted to cubbies that provide a novel stimulus is the number of bobcat captures from the nine study sites that we sampled in both 2015 and 2016. While we used the same study sites, the position of the cubbies within each resampled cell was changed to reduce the likelihood of trap shyness (Kendall et al., 2008). During the first sampling of these study sites in 2015, we recorded a total of 77 bobcat captures, as opposed to the 19 we captured in 2016—a reduction of roughly 75%. The animals we captured in 2016 at these same sites were ones that we had missed previously in 2015 or were individuals that had just moved into the area. We reached this conclusion since we did not have any multi‐year recaptures and knew from 2015 that bobcats occupied these sites. The implications of this potential lack of scent interest we observed in our study, even a year after exposure, are important for other studies using scent lures to attract bobcats to a hair snare or camera site. Reduced recapture rates caused by using the same scent lures may result in artificially inflated population estimates in research that relies on scents for bobcat sampling. Continued investigation is needed in the form of resampling the same areas beyond the lifespan of the bobcats sampled in this study to see whether capture rates are again improved.

Due to the low number of recaptures in this study, we were unable to use these data for a capture–recapture study to estimate bobcat abundance and density at our study sites. However, we constructed an occupancy model from the detection–nondetection data and estimated the minimum bobcat density of each study site (Rounsville, 2018, appendix table 1). These values were calculated from the number of genetically unique bobcats captured at each study site when considering the probability of detection. The minimum densities varied considerably between the study sites and ranged from 0 (no bobcat detections) to 15.72 bobcats per 100 km^2^ (Rounsville, 2018). When averaged statewide across both sampling years and all study sites, the calculated minimum bobcat density was 3.836 ± 0.191 bobcats per 100 km^2^ (Rounsville, 2018). Morin et al. (2018) calculated bobcat densities at study sites like ours in nearby Virginia, USA, using spatially explicit capture–recapture and found a range of 6.65–25.84 bobcats per 100 km^2^, with a mean of 12.50 bobcats per 100 km^2^. The average density of bobcats actually on the landscape in West Virginia likely lies somewhere between these two values.

### 5.1 Camera trap evaluation

Camera trapping has been successfully used to estimate bobcat population size in several studies taking place in the Midwest (Clare et al., 2015; Jacques et al., 2019), West (Alonso et al., 2015; Larrucea et al., 2007), and Southern (Heilbrun et al., 2006; Thornton & Pekins, 2015; Young et al., 2019) portions of the United States. However, in the eastern United States, where bobcats often lack distinct spot patterns on their fur coats (Croteau et al., 2012; Morin et al., 2018; Young, 1958), hair snares provide an alternative method for marking bobcats to use in population estimation (Stricker et al., 2012). While our project focused on evaluating the success of a new hair snare in the detection of wild bobcats, we did utilize some camera traps to record the interactions of bobcats with the hair snare cubby. Issues resulting from faulty trigger mechanisms on the Moultrie camera traps precluded us from conducting an objective analysis of the number of bobcats detected on camera that did or did not end up providing a hair sample. However, we were still able to collect recordings of bobcats interacting with the cubbies and have noted ways in which the sampling efficacy of the device may be improved.

Firstly, many bobcats often exhibited a rubbing behavior where they scratched their cheek scent glands on the corners of the entranceway to the cubby. Our device did not have a way to capture hairs from this behavior, and we recommend that any future studies utilize a device to collect samples from this type of interaction. Secondly, we believe that bending the gun brushes inwardly on the device may have resulted in a reduced capture efficiency. When the devices were constructed, we thought that more bobcats would choose to walk completely through the device, but this behavior was relatively uncommon. Most bobcats simply explored the cubby with their heads, and as such, some individuals did not enter far enough into the device to be sampled by the bent brushes. We recommend that future studies building on this method do not bend their gun brushes inward. Lastly, if project logistics and budget allow, the placement of additional brushes per entranceway, while still considering all brushes from a one‐week sampling at a site to be a single sample, should increase the success of capturing bobcats that interact with the device.

### 5.2 Genetic analysis

The use of smaller DNA fragments when working with samples of low DNA quantity or quality, like hair, has been correlated to amplification success and reduced allelic dropout (Broquet et al., 2006). Stricker et al. (2012) understood the importance of using small amplicon sizes for hair samples and designed primers to amplify a 250 nt amplicon of the carnivore 16S rRNA, but only successfully amplified and sequenced DNA from 56% of their total collected samples, likely due to the still large fragment size. We recommend increasing the odds of amplification success by using qPCR of small (~100 nt) species‐specific mtDNA fragments to reduce the potential for valid samples being discarded due to issues in the laboratory. However, newly developed next‐generation sequencing (NGS) species barcoding techniques will likely eclipse the usefulness of qPCR for the species identification of samples collected from hair snares (Carroll et al., 2018). This new technology can resolve mixed‐species samples and can provide species identification for all captured species, with minimal additional effort.

During the validation we completed as a part of this study, we determined that the mtDNA primer pairs of FelidV and LynX have the necessary sensitivity and specificity to determine which hair samples we collected originated from felids and bobcats, respectively. The testing we completed with DNA extracts from a panel of species that could conceivably be sampled by the cubby showed that these primers, at least in West Virginia, are specific to our intended targets. However, testing with DNA extracts from additional species located outside of our study area should be completed to evaluate the possibility of non‐specific amplification.

The sensitivity testing of FelidV and LynX that we completed revealed that samples with as little material as two small bobcat hair shafts of roughly 5 mm in length (cut from a tanned bobcat hide) were the limit of what could be reliably detected with the assay. We used the CT values of these positive controls as the bottom limit of detection for the assay and any sample that appeared positive with a CT over this threshold was considered to be negative. The sensitivity and utility of these primer pairs is most apparent in the fact that of the bobcat detections we recorded, only 60.8% were successfully genotyped. FelidV and LynX were able to provide species identification data on samples with too limited an amount of DNA for microsatellites to successfully amplify. While this may not aid capture–recapture studies where individual identifications are necessary, these detection data that would have been lost can be invaluable for habitat usage studies or occupancy modeling.

While we successfully completed our second study objective of developing a new felid‐specific qPCR method for evaluating the species of origin of collected hair samples, if we were able to complete this study again, we would use slightly different molecular methods. In our study, we concluded that samples that failed to produce PCR products for the FelidV primer set did not originate from felids. However, it is possible that either extraction failures or PCR failures resulted in legitimate felid samples being mistakenly discarded. To reduce this possibility of false negatives in our study, we extracted bobcat hair controls made from two small (~5 mm) hairs cut from a tanned bobcat pelt and completed all qPCRs in at least duplicate, including these controls. While we remain confident in our results in this study, we believe that our method could be improved by adding an additional qPCR step with a universal mammalian mtDNA primer set, such as those described in Tobe and Linacre (2008), to confirm successful hair mtDNA extraction.

## 6 CONCLUSION

The bobcat hair snare cubby represents an important advancement in creating a hair snare specifically designed for bobcats that can be deployed independent of terrain features. While our study only recaptured a small number of bobcats, we were successful in collecting samples from hundreds of wild bobcats that could be used in occupancy modeling. We believe that the hair snare cubby is a solid platform that can be improved into a valuable tool for capture–recapture studies with continued research and development. We also recommend the use of qPCR for the species identification of collected hair samples, to reduce the error caused by samples that fail to amplify or sequence with universal methods.

## CONFLICT OF INTEREST

None declared.

## AUTHOR CONTRIBUTION

**Thomas F. Rounsville:** Formal analysis (equal); Investigation (lead); Methodology (lead); Writing – original draft (lead); Writing – review & editing (lead). **Richard E. Rogers:** Conceptualization (lead); Formal analysis (equal); Investigation (supporting); Supervision (lead); Writing – original draft (supporting); Writing – review & editing (supporting). **Amy B. Welsh:** Formal analysis (equal); Funding acquisition (supporting); Investigation (supporting); Methodology (supporting); Project administration (equal); Supervision (equal); Writing – original draft (equal); Writing – review & editing (equal). **Christopher W. Ryan:** Conceptualization (supporting); Resources (lead); Supervision (supporting); Writing – original draft (supporting); Writing – review & editing (supporting). **James T. Anderson:** Formal analysis (supporting); Funding acquisition (lead); Methodology (supporting); Project administration (equal); Supervision (supporting); Writing – original draft (equal); Writing – review & editing (equal).

## Supporting information

Table S1‐S2

## Data Availability

Most of the bobcat samples used in this project were collected on private land with the permission of the landowners. We believe that publishing the exact sampling locations, especially those where bobcats were detected, will compromise the privacy of these landowners and the trust they placed in us by allowing us to sample a game species on their properties. However, the supporting microsatellite genotype data are available from the Dryad Digital Repository: https://doi.org/10.5061/dryad.3j9kd51kf.
