# Supplementary material for: Novel hair snare and genetic methods for non‐invasive bobcat detection
Source: Ecol Evol. 2022 Jan 24;12(1):e8435. doi: 10.1002/ece3.8435 (PMC8796956; doi:10.1002/ece3.8435)
Supplement: Supplementary file 1 — Table S1‐S2 [file ECE3-12-e8435-s001.docx]

Supplemental Table 1. Bobcat microsatellite population genetics data was calculated as a part of Rounsville (2018) using the program CERVUS v.3.0 (Kalinowski et al. 2007). These data were generated from the genotype profiles of 280 individual West Virginia (USA) bobcat spleen samples collected during necropsy between November 2014 through February 2015 (Landry 2017). The column headings are as follows: k is the number of unique alleles at a locus, H_O_ is the observed heterozygosity, PIC is the polymorphic information content, PI is the combined non-exclusion probability of identity, and Psib is the combined non-exclusion probability of identity for siblings.

| Locus | k | H_O_ | PIC | PI | Psib |
| --- | --- | --- | --- | --- | --- |
| FCA90 | 9 | 0.845 | 0.811 | 0.050 | 0.346 |
| FCA23 | 10 | 0.716 | 0.737 | 0.086 | 0.386 |
| FCA26 | 13 | 0.645 | 0.633 | 0.149 | 0.448 |
| 6HDZ057 | 11 | 0.702 | 0.545 | 0.216 | 0.504 |
| FCA008 | 15 | 0.849 | 0.757 | 0.076 | 0.375 |
| FCA77 | 15 | 0.706 | 0.788 | 0.060 | 0.359 |
| FCA43 | 15 | 0.736 | 0.631 | 0.148 | 0.452 |
| 6HDZ700 | 11 | 0.736 | 0.643 | 0.136 | 0.448 |
| FCA45 | 13 | 0.777 | 0.676 | 0.124 | 0.419 |

Supplemental Table 2. Pairwise non-exclusion probability of identity (PI) / pairwise non-exclusion probability of identity for sibling bobcats (Psib) at the microsatellite loci of FCA008, FCA77, and FCA90. Bobcat microsatellite population genetics data was calculated as a part of Rounsville (2018) using the program CERVUS v.3.0 (Kalinowski et al. 2007). These data were generated from the genotype profiles of 280 individual West Virginia (USA) bobcat spleen samples collected during necropsy between November 2014 through February 2015 (Landry 2017).

| Locus | FCA008 | FCA77 | FCA90 |
| --- | --- | --- | --- |
| FCA008 | - | 4.56 x 10^-3^ / 0.135 | 3.80 x 10^-3^ / 0.130 |
| FCA77 | - | - | 3.00 x 10^-3^ / 0.124 |
| FCA90 | - | - | - |
